# An Enhanced Ant Colony Optimization Mechanism for the Classification of Depressive Disorders

**DOI:** 10.1155/2022/1332664

**Published:** 2022-06-28

**Authors:** Abed Saif Alghawli, Ahmed I. Taloba

**Affiliations:** ^1^Computer Science Department, Prince Sattam Bin Abdulaziz University, Aflaj, Saudi Arabia; ^2^Department of Computer Science, College of Science and Arts in Qurayyat, Jouf University, Sakaka, Saudi Arabia; ^3^Information System Department, Faculty of Computers and Information, Assiut University, Assiut, Egypt

## Abstract

Bipolar disorder is marked by mood swings that alternate between mania and depression. The stages of bipolar disorder (BD), as one of the most common mental conditions, are often misdiagnosed as major depressive disorder (MDD), resulting in ineffective treatment and a poor prognosis. As a result, distinguishing MDD from BD at an earlier phase of the disease may aid in more efficient and targeted treatments. In this research, an enhanced ACO (IACO) technique biologically inspired by and following the required ant colony optimization (ACO) was utilized to minimize the number of features by deleting unrelated or redundant feature data. To distinguish MDD and BD individuals, the selected features were loaded into a support vector machine (SVM), a sophisticated mathematical technique for classification process, regression, functional estimates, and modeling operations. In respect of classifications efficiency and frequency of features extracted, the performance of the IACO method was linked to that of regular ACO, particle swarm optimization (PSO), and genetic algorithm (GA) techniques. The validation was performed using a nested cross-validation (CV) approach to produce nearly reliable estimates of classification error.

## 1. Introduction

**Figure 1:**
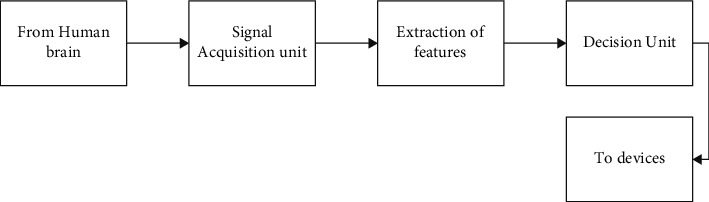
EEG signal processing.

Information science and data capture systems have made it easier to collect and store large datasets using extended time series. These datasets are becoming more and more popular in a variety of sectors, including astronomy, environment, economics, business, medicine, data analysis, and knowledge mining [1]. The assessment of large datasets is a significant activity in and of itself, and current studies highlight the usage of FS approaches and their talented findings. Seizure identification, sleep state identification, and activity recognition categorization are just a few of the applications where digital analysis of EEG signals is useful. As shown in Figure 1, digital EEG signal treatment consists of three components: a signal acquisition device, an extraction of features unit, and a decision mechanism. The EEG signal collected from a scalp, brain surfaces, or brain internal represents the service's input in Figure 1. Electrodes, either invasive or noninvasive, are used to indicate the signal acquisition component [2]. The extraction of features unit seems to be a signal processing component that extracts distinguishing features from such a stream (*s*). In a brain-computer interface (BCI), for instance, the decision section is a hybrid component that performs classifications, decision-making, and transmitting choices to remote systems that reflect the subject's desire [3].

Metaheuristic algorithms are often described as combining probability, randomness, and mathematical notation to mimic usual processes. Biological evolutionary processes such as the genetic algorithm (GA) and differential evolution (DE), behavioral genetics such as ACO and particle swarm optimization (PSO), and physically hardening processes such as evolutionary algorithms are examples of these processes (SA) [4]. In recent research, several metaheuristic algorithms and enhanced versions have been effectively applied to a variety of optimization situations. Those techniques outperformed traditional mathematical solutions in terms of generating better results. ACO is a random optimization approach based on empirical evidence of social behaviors of real animals or insects, but it is an effective algorithm for FS issues between these metaheuristic techniques. Nevertheless, ACO still has several shortcomings in practice. The algorithm's primary criteria are the possibility of becoming caught in a local optimum, the high computing effort and system resources required to get the best solution, and the difficulty of setting the heuristic method to attain high efficiency [5]. As a result, similar enhanced ACO approaches have been developed in recent studies to minimize such faults.

As a result, categorizing MDD and BD at an earlier stage of illness may aid in more efficient and targeted treatment. Neuroimaging approaches for BD and MDD were employed in certain research to demonstrate distinct patterns of structural and functional brain alterations in brain systems involved in emotion regulation [6]. Statistical inference methods were employed in several other investigations, and because those approaches rely solely on the concept of linear combinations, they may not be suited for such assignments. Machine learning approaches have become increasingly popular in the research of anxiety symptoms and comparing these individuals to others with different psychiatric diseases over the last decade. SVM was applied in a recent study to examine the detection accuracy of MDD and BD, and the participants were categorized with an accuracy of 54.76% [7].

Information processing analysis was used in a similar investigation with compartments of the anterior cingulate cortex (ACC) blood circulation in repose. SVM diagnosed MDD and BD patients with 81% accuracy using subgenual ACC blood flow [8]. Another study used multidimensional pattern classification methods and brain morphologic indicators to differentiate the two sad groups with up to 79.3% accuracy. In that other work, the automated identifying technique is used to predict epileptic seizures in multichannel EEG recordings. For feature extraction, approximated probability and statistic estimates were used for both MDD and BD EEG recordings. The effectiveness of SVM with different functions of kernel in detecting seizures is also investigated in this work utilizing MDD and BD EEG signals. The RBF kind kernel SVM algorithm has a predictive performance of 97.17% [9]. Aside from their contributions to diagnosis through great classification accuracy, computerized feature extraction approaches are increasingly being used in the research of anxiety symptoms and assessments of psychiatric disorders. With such a lifetime frequency of up to 4-5%, depression episodes in BD are considered one of the most common psychiatric diseases. While MDD and BD are treated as separate clinical situations with different therapeutic approaches, previous studies found that 60%  of BD situations were misdiagnosed as UD and treated wrongly as a result [10].

Consequently, identifying biomarkers that indicate unique pathophysiologic mechanisms in BD and MDD is vital. One of the possible neurophysiological indicators for brain waves is EEG consistency. Different spatial consistency gestures were gathered across large distances as parallelization, and coherence represents the interaction among signals in a certain frequency range [11]. EEG coherence is a unique large number of functional linkages coordinated effective among cortical region pairings, making it a biomarker for the brain's functional connectivity. The clinical importance of cohesiveness as a biomarker in the identification of psychiatric diseases has been highlighted in recent research. Using SVM and IACO, this research intends to expose the distinguishing features of MDD and BD individuals without compromising their accuracy of classification [12]. The levels of coherence, an indicator of functional connectivity within a brain, were first determined utilizing a method that had been previously identified.

Section 2 explains previous work related to this research, and Section 3 lists the topic, EEG data, and procedures for calculating coherence.  Section 3.4 describes the FS approach and a hybrid architecture with SVM.  Section 4 presents and discusses the results of experiments and validation of performance with current work. Finally, Section 5 concludes the study.

## 2. Related Work

Motor activity has been the most constant sign of bipolar disorder, according to a growing body of scientific research. Body position, cognitive responsiveness, degree of the psychomotor action, and speech-related motor functions are all examples of motor activity. Self-reported questions with medical observer-rated scales have traditionally been employed in investigations of motor activity in bipolar disorder, which are sensitive and often ineffective. Motor activity data could be used to define phase kind in bipolar patients, which is important because severe sadness and manic episodes can be fatal. This research describes a system that uses sensing data on phones to identify the status of bipolar illness sufferers. Use real-life activities, collected voice, accelerometer, and self-assessment information from 5 patients more than 12 weeks [13]. The effectiveness of numerous classifiers, the distinct sets of characteristics, and the role of questions in categorizing bipolar disorder events were assessed in this study. This has already shown, for example, that the trajectory of mood disturbances or recurrence in bipolar patients can be classified with high confidence (≈85%). To our knowledge, no study has concentrated on observational research of everyday phone conversations to diagnose reduced life performance in bipolar disorder patients. But, there are also plenty of other semisupervised techniques that could be used to enhance such performance in the future [14].

Major depressive disorder (MDD) is the important source of blindness and mortality, affecting around 10% of people worldwide. There are currently no clinically effective diagnostic indicators that can distinguish MDD and bipolar disorder (BD) in an early stage of a period of depression. Exploring machine learning-based integrative indicators of mood disorders is thus an urgent, albeit difficult, need with significant promise to increase the knowledge of such disorders. In this study, we look at some of the most common machine learning algorithms for brain scan prediction and classification, as well as a summary of research that used diagnosis methods imagery information to differentiate MDDs from normal as well as other mood disturbances and look into individual patient diagnostic accuracy determinants. Finally, problems, future trends, and potential limits linked to MDD biomarker development are reviewed, to provide a thorough overview that will help readers in understanding how neuroimaging data gathering might be used to treat depression. Thus, we believe that such efforts would draw attention to the critical want for a medical paradigm change that will drive tailored, optimal patient care [15].

Due to the lack of identified biomarkers, distinguishing between bipolar disorder (BD) and major depressive disorder (MDD) is a significant medical difficulty; thus, a deeper knowledge of their aetiology and brain abnormalities is urgently required. In neuroimaging applications, feature selection is extremely critical due to its intricacy; yet, feature dimension and model understanding are significant issues. A wrapper feature selection method is proposed based on the forward selection method (SVM-FoBa) and used to collect anatomical and resting-state functional magnetic character imaging information from 21 BD, 25 MDD, and 23 healthy controls in this investigation. Discriminative characteristics were extracted between both data modalities, and the categorization of MDD and BD was 92.1% accurate. In adding to the default mode networks and the cerebellum, value assessment of the features extracted suggested that the inferior frontal gyrus, along with the default mode system and the cerebellum, may play a significant role in BD-MDD distinction. When identifying the two clinical illnesses, functional information trumps biological information by a considerable margin, according to a modality-by-modality comparison. This study confirmed the benefits of multimodal collaborative efforts and the efficacy of SVM-FoBa that has the potential to be used to detect a potential biomarker for a variety of mental diseases [16].

Human-computer intelligent interaction (HCII) is a growing branch of study that strives to improve and develop human-computer interaction. As a result, several research works have been carried out to identify human emotion using various techniques including facial expression, voice, galvanic skin response (GSR), and heart rate (HR). However, such strategies have issues with reliability and validity, because people can lie about their feelings and responses. The electroencephalogram (EEG), on the other hand, has proven to be a very successful method of identifying people's emotions since it captures human brain function, which cannot be tricked by voluntary control. Despite the approval of EEG in identifying human reaction, research in this subject is difficult since the EEG signal is nonlinear, complex, and disordered. As a result of these concerns, there is a high-dimensional feature problem with poor classification performance [17]. This paper proposes a novel computational methodology to solve such issues, which consists of three basic stages: (a) extraction of features, (b) feature selection, and (c) classification. To obtain EEG signal characteristics, the discrete wavelet packet transform (DWPT) was utilized, and 204,800 features from 32 subject-independent subjects were acquired. Meanwhile, as a feature selection method and classification, the genetic algorithm (GA) and least squares support vector machine (LS-SVM) were applied. This computer model is used to distinguish three levels of emotion and attentiveness using the typical DEAP preprocessed EEG dataset. The suggested GA-LS-SVM increased the classification performance to 49.22%  and 54.83%  for emotion and excitation, respectively, whereas when no feature selection strategy was applied, the classification performance was 46.33 percent and 48.30 percent for emotion and excitation, respectively [9].

With technological advancements, we now have new options for the long-term management of health issues. In psychiatry, there are numerous chances where the diagnosis is based on the patient's historical information as well as their current emotional responses, which adds to the difficulty of differentiating between bipolar illness and borderline disease after analysis. This publication was influenced by previous research that classified illnesses by treating symptoms as a time sequence phenomenon. This work presents a signature-based machine learning strategy for extracting distinctive temporal patterns that can be associated with a given illness. One of the primary criteria used to determine the disorder in this approach is the consecutive quality of the information. This study looks at examples of borderline syndrome which are either biologically passed on from parents or arise from childhood effects of extreme fear and stress. The model is validated using the Alan Turing Institute synthesized signature database within pattern psychiatric repository. The end outcome has an AUC of 0.95, which is higher than the previous result of 0.90 [18].

## 3. Materials and Methods

### 3.1. Topics

Consider the patients who registered to a Hospital Department of Psychiatric Outpatient Clinic for Neuropsychiatry and underwent a retrospective assessment. This study included 101 patients who were diagnosed with BD and MDD at the time of their admission. A total of 48 bipolar disease patients (18 men and 30 women) and 58 MDD participants (25 men and 33 women) were compared [19]. Outpatients with a period of depression of BD or MDD who were identified on the Individual Examination Meeting for Axis I Disorders as per the Diagnostic and Statistical Manual of Mental Disorders (DSM)-IV criteria for any a initial analysis of bipolar effective long-term disorder depressive incident or major depressive disorder (SCID-I) were selected.

Individuals with a diagnosis of MDD and a value of at least 8 here on Hamilton Depression Rating Scale (HDRS) 17-item form or focuses with an analysis of the BD depressive episodes and a score with at most 13 here on Young Mania Rating Scale (YMRS) were included. Subjects who had their first period of depression, a chapter with present psychotic features, a background of rapid cycling, a history of mixture occurrences, existing psychiatric comorbidity on axis I, a serious unstable medical illness or neurocognitive disorder, or alcohol and substance violence within the previous 6 months and patients hospitalized for electroconvulsive therapy within the previous 3 months were excluded from the study [20]. Individuals with fewer than four psychiatric admissions were also excluded. This factor was established to assure the diagnosis's long-term consistency. All of the patients were assessed by four psychiatrists with at least five years of clinical experience. There was no assessment of interrater reliability. Subjects with fewer than four healthcare practitioners were also selected. This criterion was established to assure diagnosis consistency throughout time. Out of caution, none of the patients was taking antidepressants at the moment of the EEG recording. QEEG recording was once a standard practice performed before treatment decisions for individuals who sought therapy at a neuropsychiatry hospital. BD patients who were given a mood preservative or a combination of mood stabilizers, on the other hand, had a better outcome. At research screening, individuals had to pass normal laboratory tests, a urine toxicology test, and an electrocardiogram, and they had to be physically steady before enrolling in the research.

### 3.2. EEG Recording Data

EEG was obtained for 5 minutes in an eyes closed sleeping steady state for all the individuals. Patients were told not to take any medicine for 12 hours well before EEG recording. Qualitative EEG (QEEG) data are gathered from 101 people who were sat in a sound-attenuated, electronically insulated environment in a chair in the living room with eyes closed (waking state situation) to monitor and show the efficiency of coherence [21]. To avoid sleepiness, the technicians maintained the QEEG information during the recordings and re-altered the patients every minute as necessary. In accordance with the international 10/20 system configuration, 19 taped electrodes were implanted over the skull.

**Figure 2:**
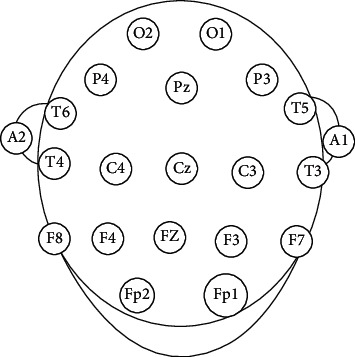
EEG electrodes.

The scan LT EEG amplifiers and electrode caps were used to record 3 minutes of eyes closed EEG during rest at a sampling frequency of 250 Hz. An array of 19 sintered Ag/AgCl electrodes were positioned to give a 10/20 international organization signal. From Figure 2, intrahemispheric synchronization was evaluated on the left hemisphere using electrode pairs *F*3–*C*3*F*3–*P*3, *F*3–*T*5, *C*3–*P*3, *C*3–*T*5, *P*3–*T*5 and then on the right hemisphere using electrode pairs *F*4–*C*4, *F*4–*P*4, *F*4–*T*6, *C*4–*P*4, *C*4–*T*6, *P*4–*T*6. Electrode pairs *F*3–*F*4, *C*3–*C*4, *P*3–*P*4, and *T*7–*T*8 were used to evaluate interhemispheric synchronization. Before artifact removal, the raw EEG data were filtered using a band-pass filter (0.15–30 Hz) and EEG sections with an evident eye and head, and muscular artifacts were carefully eliminated.

### 3.3. QEEG Biomarkers

The magnitude-squared synchronization function, which is dependent on the Fourier series of the frequencies, *f* values, was used in traditional EEG spectral analysis. The normalized frequency response per wavelength of two frequencies collected simultaneously at distinct places on the scalp is described as synchronization [22]. For each combination of electrodes, total magnitude-squared coherence *H*_*xy*_(*f*) is computed as squares of exponent of average cross-power spectral density (PSD) regularized to a product of the mean auto PSD. The coherent frequency for the waveform *x* and *y* of an electrode's combination is determined as

(1) $$\begin{array}{c} H_{xy}\left(f\right)=\frac{{\left|P_{xy}\left(f\right)\right|}^{2}}{\left|P_{xx}\left(f\right)\right|\left|P_{yy}\left(f\right)\right|}, \end{array}$$

where *P*_*xy*_(*f*) denotes the cross PSD approximate of *x* and *y*, while *P*_*xx*_(*f*) and *P*_*yy*_(*f*) denote the PSD estimations of *x* and *y*, respectively. The cross-power spectra and the power spectrum (wide range of measures) are described as

(2) $$\begin{array}{c} P_{xy}\left(f\right)≔{\left|x^{\prime \prime }\left(f\right)\right|}^{2}=x^{\prime \prime }\left(f\right)\bar{x^{\prime \prime }\left(f\right)},\\ P_{xy}\left(f\right)≔x^{\prime \prime }\left(f\right)\bar{x^{\prime \prime }\left(f\right)}, \end{array}$$

where *x*^″^ is a complex conjugate of the *x*:

(3) $$\begin{array}{c} x^{\prime \prime }\left(f\right)≔\int_{-\infty }^{\infty }x\left(t\right)e^{-iwt}\text{d}t. \end{array}$$

Equation (3) is the Fourier transform which provides data on the frequency which occurs in signals as well as their dominant frequency. The raw EEG signals were split into 650 ms intervals with a 50% overlap, but the settings of every period are displayed using a Hanning window even during the calculating process. The MATLAB application was then used to perform coherent analyses of each participant using 10–14 artifact-free periods [21]. Among the targeted and non-targeted activation situations, the scan frequencies were chosen at random. For long-range intrahemispheric and interhemispheric couples in the delta Δ, theta *γ*, and alpha *α* radio frequencies, coherence standards are determined for such target and non-target stimulation. Then, Fisher's *Z* conversion was utilized to standardize the distributions of coherence levels.

### 3.4. Phase of Feature Selection

This paper offers an FS stage to handle the large dimensional feature storage problem, in which a sample of important characteristics is picked to improve classification performance. In this research, a genetic algorithm is used with an LS-SVM classification [23]. GA is a multidimensional problem-solving technique that, similar to gradient search approaches, avoids the problem of local optima. Furthermore, the GA algorithm has a broad and deep search space exploration capability. This method is inspired by the natural evolving criteria mechanism, which was created to emulate biological behavior to optimize a complex objective function. The optimization was simply achieved by allowing a community composed of many people to progress under specified and specific restrictions to advise that maximizes actual health. GA is used in this research under the MATLAB programming framework. The wrapper decision is built using GA and LS-SVM classifier algorithms [24]. Individuals within a population were assigned binary strings 1 and 0, indicating whether the feature was taken into account and whether it was included or not. However, the classification accuracy is defined also as a fitness function.  Table 1 provides how the GA algorithm's choices variables were set up in this investigation.

The FS process is a widely used approach for reducing data dimensions and improving the effectiveness of the learning algorithms. For an FS procedure, the entire search space encompasses all potential subsections of features, and the degree of subsections is computed using the following equation:

(4) $$\begin{array}{c} \overset{x}{\underset{z=0}{\sum }}\left(\frac{x}{z}\right)=\left(\frac{x}{0}\right)+\left(\frac{x}{1}\right)+\cdots \left(\frac{x}{x}\right)=2^{x}, \end{array}$$

where *n* denotes the number of characteristics in the existing subset of features and *z* is the size of the entire feature subset. FS approaches typically necessitate heuristics or randomized search tactics, which add to the complexity of the resulting group, lowering its degree of optimization problem [25]. Depending on their evaluation approach, FS techniques can be divided into three categories. A filter method is when a procedure conducts FS independently of a learning algorithm, and it usually involves picking feature subsets according to the interclass conditional independence principle. The filter technique is popular for working with high-dimensional data because of its processing efficiency. When a classification method is used to conduct the evaluation stage, the FS method is referred to as a wrapper method.

**Figure 3:**
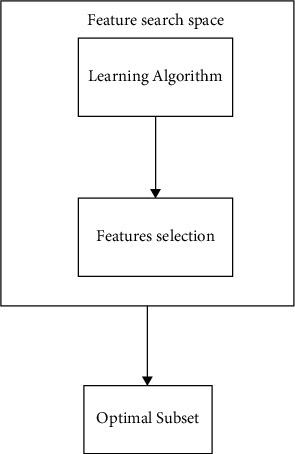
Wrapper-based approach process.

In a wrapper method, selected structures are placed into a preprogrammed learning study to estimate the subset's effectiveness. In comparison to filters, the prediction accuracy of final specific subset in wrappers is connected with specified relevance measure; however, trade with a large dataset could grow the difficulty owing to the employment of learning techniques in feature subset assessment. To conclude, similarly to wrapper techniques, the FS and learning algorithms are interspersed within the embedded method, and the link between the FS and the classification is deeper; however, wrapper methods cover more of the search space [26]. As shown in Figure 3, wrappers are composed of three main components: a learning machine, feature assessment methods, and an FS technique. Metaheuristic search approaches appear to be significant due to their versatility in randomized searches to assist with the FS procedure since a large selection of attributes may generate high complications. Nature-inspired techniques including genetic algorithm (GA)-based attribute reductions, particle swarm optimization (PSO), and gravitational search algorithm (GSA) are highlighted in recent publications. Besides these techniques, which aim to improve solutions by applying knowledge from earlier iterations, ACO seems to be another promising method for solving combinatorial optimization issues, and it has been frequently used in FS.

### 3.5. Feature Selection with an ACO Algorithm

**Figure 4:**
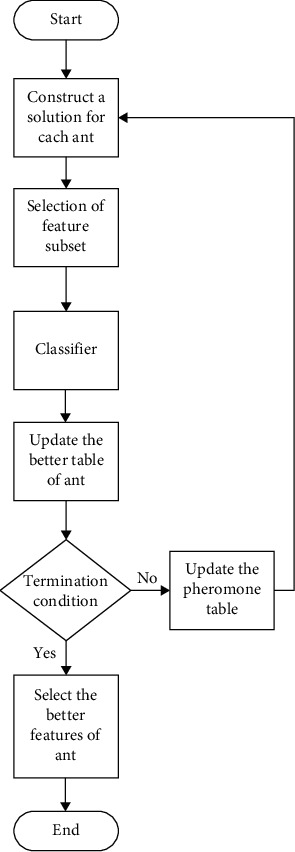
Proposed design of ACO-based parameter of feature selection.

In pattern recognition problems, feature selection and dimensional reductions are critical phases. Even though the set of features was not extensive in this investigation and the results were adequate, utilizing the most significant attributes improved the classification performance [27]. The classifier was also able to develop a more efficient approach and obtain higher generalization performance by reducing the number of characteristics. The ACO method was used to obtain an optimal subset of characteristics, and the flowchart of the optimization procedure is shown in Figure 4.

The optimization method and the BPNN classifiers are used to perform the feature extraction optimization processing steps. ACO's chosen features are transmitted to the classifiers, and the resulting model is evaluated with such a test set containing the allocated features. Lastly, the MSE loss is used to assess every ant's performance to modify a pheromone database. The procedure continues to meet the halting criteria, which is specified as a standard error that is as low as possible. ACO is a stochastic metaheuristic for solving complex optimization problems that are continuous. It is inspired by the foraging strategy used by real biological ants when they are trying to discover a simple path between their colony and a source of food [28]. The ants interact informally while hunting using pheromones to indicate their pathways and attract additional ants. Artificial ants produce simulated pheromones to modify their route across the decision network, which would be the route that indicates which alternative nodes an agent will choose, in the ACO algorithm. The volume and intensity of pheromones that the agent utilizes to modify its path are determined by how effective the response is when compared to the previous iteration's competitor species. While making their own decisions to determine the best route of all feasible possibilities, the ensuing representatives employ the pheromone markers of preceding good agents as a reference.

The ACO was initially used for the optimization of the traveling salesman issue because it resembled taking the shortest route to a food supply [29]. A set of cities (nodes) is supplied in this problem, and the distances between them are known. The goal is to determine the quickest route that permits you to visit each city only once. Alternative routes are developed using a probabilistic model, and these trails are stated to build by the artificial ants moving across a network that describes the problem, with each vertex representing a town and every edge representing a connection among two cities, according to the ACO metaphor. Attempts to construct an ACO algorithm were unsuccessful again until the technique was combined with local optimization. One issue is early converging to a suboptimal solution due to the rapid application of too much synthetic pheromone. Pheromone absorption is used to solve this issue. To put it another way, the pheromone linked with solution vanishes later after a certain amount of period. Ants use a stochastic process to choose the next city to visit in the creation of a solution. Assuming ant *r* is in city *p* and has completed the partial equation *T*_*s*_, the chance of it traveling to city *q* is

(5) $$\begin{array}{c} S_{pq}^{r}=\left\{\frac{\tau_{pq}^{\sigma }.\eta_{ps}^{v}}{\sum_{C_{ps}}\in N\left(T^{s}\right)\tau_{pq}^{\sigma }.\eta_{ps}^{v}}\right.,\;\text{if}\;C_{ps}\in N\left(T^{s}\right), \end{array}$$

where *N*(*T*^*s*^) is the quantity of possible nodes, which would be provided as follows: *σ* and *v* are the parameters to regulate the relative relevance of the pheromone's vs. the heuristic input *η*_*pq*_.

(6) $$\begin{array}{c} \eta_{pq}=\frac{1}{d_{pq}}, \end{array}$$

where *d*_*pq*_ is the distance between *p* and *q*.

During every phase, all of the effective and efficient which have built responses in that iteration modify the pheromones quantities. The following is an update to the pheromone *τ*_*pq*_, which is connected with the edge connecting cities *p* and *q*:

(7) $$\begin{array}{c} \tau_{pq}\leftarrow \left(1-\rho \right).\tau_{pq}+\sum_{r=1}^{m}\eta \tau_{pq}^{r}, \end{array}$$

where Δ*τ*_*pq*_^*r*^ is the volume of pheromones placed on edge (*p*, *q*) by ant *r*, *ρ* is also the evaporation speed, and *m* represents the number of ants.

Here,

(8) $$\begin{array}{c} \Delta \tau_{pq}^{r}=\left\{\begin{array}{cc} \frac{\delta }{Sq}, & \text{if ant used edge}\left(p,q\right),\\ 0, & \text{otherwise,} \end{array}\right. \end{array}$$

where *S*_*q*_ is the duration of a tour built by ant *q*, and *δ* is a variable.

### 3.6. Support Vector Machines

A training set of values is given as $Z=\left\{\left(p_{i},q_{i}\right)|a_{i}\in H,q_{i}\in \left\{\begin{array}{c} +\\ - \end{array}1\right\},\;i=1,2,\ldots ,s\right\},$ where *p*_*i*_ represents the input variables and *q*_*i*_ represents the *x*_*i*_ label; the objective purpose is determined as

(9) $$\begin{array}{c} \left\{\begin{array}{cc} \min\varphi , & \left(W\right)=\frac{1}{2}W.W+C\sum_{i=1}^{s}\delta_{i},\\ \text{subject to,} & q_{i}\left(W.\varphi \left(x_{i}\right)\right)+b\geq 1-\delta_{i},\delta_{i}\geq 0\;i=1,2,\ldots ,s, \end{array}\right. \end{array}$$

where *W* is the hyperplane normal variable and *C* is a penalty ratio that regulates the trade-off between maximization of a valuation width and minimization of the number of misidentified instances in the training collection to 10 [30]. The width of the kernel is controlled by the hyperparameter *δ*_*i*_ which is set at 0.2. Lastly, the ideal hyperplane is written as the quadratic function in the following equation:

(10) $$\begin{array}{c} \left\{\begin{array}{c} \max S\left(\alpha \right)=\sum_{i=1}^{s}\alpha_{i}-\frac{1}{2}\underset{ij}{\sum }\alpha_{i}\alpha_{i}q_{j}q_{j}S\left(p_{i}p_{j}\right),\\ \text{subject to}\sum_{i=1}^{s}\alpha_{i}\;q_{i}=0,0\leq \alpha_{i}\leq C,\;i=1,2,\ldots ,s. \end{array}\right. \end{array}$$

The output data can be stated in the following equation as

(11) $$\begin{array}{c} f\left(p\right)=\text{sign}\left[\sum_{j=1}^{s}\alpha_{i}q_{i}S\left(p_{i},p\right)+b\right]. \end{array}$$

Different kernel functions might be employed in the decision function based upon the data. The most commonly used kernel functions include polynomial, radial basis function (RBF), and linear, which are all listed in the following equation:

(12) $$\begin{array}{c} S\left(p,p_{i}\right)=\exp\left(-\frac{p-p_{i}^{2}}{2\sigma^{2}}\right). \end{array}$$

In this investigation, 3 kinds of kernels have been used, and the RBF kernel was chosen because of its superior performance, as given in Table 2.

### 3.7. IACO-SVM Proposed Techniques

In the proposed method, IACO processes the inputs as a specified feature subset, while the outputs are categorized as psychiatric illness types. To accomplish this, all characteristics of alpha, delta, and theta coherence values are given in the FS step, and more relevant characteristics are picked using a metaheuristic approach involving artificial ants [31]. The fitness value well over the SVM classifier can then be utilized to evaluate the performance of the various feature subsets. The pheromone updating process is started based on the fitness value, and heuristic parameters are changed as a result. The modeling procedure repeats itself until the halting requirement is met. The classification performance is evaluated after each modeling session using exterior testing data which is novel to the system.

A nested-CV is made up of two-layered cross verifications, exterior one and interior one. To begin, the dataset is divided into six pieces, each with a 6-fold outer-CV and sampling methods. Varies from one person fold is set aside for the outer-CV test, the remaining 5 folds are saved for the inner-CV cycle's training phase. A 5-fold CV method is used in the inner-CV cycle, and 5 models are generated in the end [32]. The classification performance of the models using the specified subsets of features was then ordered. The greatest of those 5 models is evaluated using restricted outer-CV assessment folds which are totally unfamiliar to the classifiers to minimize leaking from testing and train the model.

During the interior CV loop, the conserved outer-CV test folds are exchanged with a few of five training folded, allowing the inner-CV models to be tested with entirely different test data for every external CV cycle. Inner-CV training data is used to identify features utilizing IACO in internal CV. Four folds are utilized for training and one for verification in the IACO method. The aforementioned procedures are performed five extra times to obtain the local best system for each loop, as the data was separated into six pieces in outer-CV. Last, the arrays are sorted by classification results to find the globally highest of six regionally best systems with their optimized subset of features.

### 3.8. Fitness Value

The level of usefulness of a specific subset is calculated using a fitness function. If two subgroups with various numbers of characteristics perform similarly well in a classification issue, the subset with the fewer characteristics takes the lead [33]. As a result, the fitness value is evaluated in terms of two factors: classification accuracy and the number of components in the subset. To address these problems, the fitness value is meant to be as accurate as possible while also having a large variety of features in the following equation:

(13) $$\begin{array}{c} f\left(a_{j}\right)=m^{*}J\left(A_{j}\right)+n^{*}\left(\frac{1}{\left|A_{j}\right|}\right), \end{array}$$

where *A*_*j*_ is the *j* ^th^ ant's subset, *J*(*A*_*j*_) is the classifying accuracy utilizing (*A*_*j*_), |*A*_*j*_| is the variety of attributes in *A*_*j*_, and *m* ∈ [0,1] and *n* ∈ [0,1] are two variables used to provide relative priority to organization performance and the number of subset strictures. Since classification performance is crucial in comparison to the number of characteristics, we selected *m*=0.92 and *n*=0.78 in the analysis. The fitness values are computed by replacing the coefficients within a solution, as given in Table 3.

### 3.9. Analysis of Complexity

#### 3.9.1. Time Complexity

To construct a model with fewer but more useful features, the hybrid strategy integrating IACO and SVM algorithms was used in this research [34]. Using the values in (14), the computational difficulty of the suggested method in terms of time may be calculated:

(14) $$\begin{array}{c} N_{\text{iter}}\times \left(N_{\text{Ants}}\times \left(T_{\text{selection of features}}+T_{\text{SVM}}\right)+T_{\text{Updating the pheromne}}\right). \end{array}$$

The number of iterations is *N*_iter_, while the number of ants within every iteration is *N*_Ants_. *T*_selection of features_ is the time it takes an ant to produce a subset of features, *T*_SVM_ learning is the time it takes an ant to train an SVM classifier using selected features, which expands under a particular (*O*(*n*^3^)) with the samples in the training set (*n*), and *T*_Updating the pheromne_ is the time it takes an ant to upgrade the pheromone table after a model has been produced.

**Figure 5:**
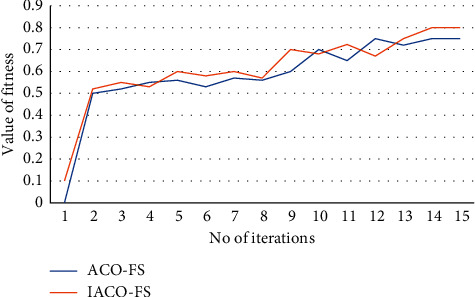
With different numbers of iterations, the ACOFS and IACOFS techniques have different fitness values.

The fitness value calculation method, described in (13), is performed for all iterations, as shown in Figure 5. The effectiveness of the methods improves as the number of iterations grows. The convergence efficiency of both the ACO and IACO approaches was investigated by running the algorithms ten times [35–37]. In Figure 5, the average values of the minimal fitness functions of those 10 sessions are shown against the number of iterations. It is clear that the suggested method, IACO, converges faster and more accurately than the classic ACO algorithm. In addition, 20 trials were conducted to evaluate the average execution duration of regular ACO, PSO, GA, and IACO, the outcomes of which are given in Table 4.

### 3.10. Complexity of Space

A system's space complexity is measured by the amount of memory used across the entire procedure. The algorithm's memory footprint is estimated utilizing both fixed and variable storage. The variables utilized in the program take up a set amount of space, whereas the component whose size is determined by repetitions and recurrent operations takes up a variable amount of space [38–40]. Two space characteristics are taken into account while determining the overall amount of storage used against the algorithm. Factors, data structures, allotted memory, and other data components are used to describe the quantity of subspace. The space difficulty is determined as in the following equation, where the magnitude of the challenge is *m* and the number of ants is *n*:

(15) $$\begin{array}{c} S\left(m\right)=S\left(m\right)=O\left(4m^{2}\right)+O\left(2mn\right)\text{.} \end{array}$$

Because the issue dimension should be the same for IACO and simple ACO, the case complexity will be the same.

## 4. Result and Discussion

**Figure 6:**
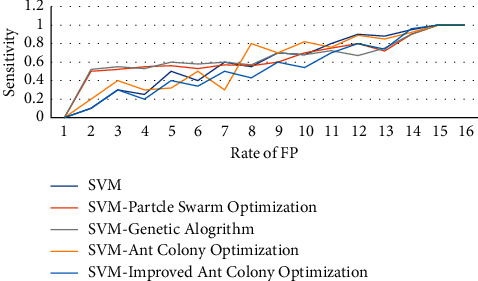
The effectiveness of each technique.

A series of tests are carried out in this work to demonstrate the efficacy of the suggested FS method. SVM with a selection of features extracted was used to classify 46 BD and 55 MDD cases. The genetic algorithm (GA), particle swarm optimization (PSO), and ACO and IACO algorithms were used in the FS process. The heuristics variables for IACO were changed during the optimization phase to mitigate general ACO limitations such as earlier stagnation and slow convergence speed. Twelve interhemispheric and four intrahemispheric QEEG synchronization measures from alpha, delta, and theta bandwidths make up the initial feature set. To train and test the SVM classifier, nested-CV was used. This used ROC and area under the curve (AUC) analyses for every combined and standalone classification model because ROC analysis is a useful tool for displaying how classifications and threshold selections work.  Figure 6 shows the effectiveness of each technique.

The suggested approach, IACO-SVM, improves GA-SVM, PSO-SVM, ACO-SVM, and standalone SVM classifiers, as shown in the figure. The number of characteristics, accuracy rate, sensitivity, and AUC levels of classifiers with different FS approaches are given in Table 5 to stress the importance of dynamic heuristics utilized in IACO.

The efficiency, sensitivity, and AUC ratings of the standalone SVM classifier are not sufficient, even though it has more parameters than classifiers using FS techniques. With limited parameters, assigning FS techniques to the classifier improved classification results by 73.26%. According to IACO's actual performance, variable heuristics variables boost the accuracy of classification better. As a result, we improved regular ACO and saw a significant rise in both total classification results and AUC value, highlighting the necessity of giving relevant attributes to a model.  Table 6 also includes the specific characteristics that lead to the classification results, as well as a visualization of the resulting extracted features using Table 6.

Only a few studies have looked into the differences in brain connectivity between MDD and BD in terms of clinical translation of a processing variable in the IACO-SVM model used in this research. Using univariate approaches, research group discovered a paucity of frontal interhemispheric alpha connection in BD patients in comparison to MDD patients in such a new analysis. The study found that BD patients had more alpha activity in the contralateral temporoparietal areas than UD patients. Furthermore, a study conducted argued that the absence of interhemispheric synchronization in the slow-wave frequency ranges could be one of the characteristics distinguishing BD from UD. In contrast to the previous studies, delta frequency range was included in the results.

The explanation seemed to be that the delta frequency range was linked to cognitive performance, which was found to be worse in bipolar disorder patients than in those with significant depression. Second, specific changes in the delta band were observed during the sleep of depressive individuals, suggesting that the delta band may be used as a control band because recordings were taken in an eyes closed, nonsleep state. This could explain why delta frequency contributed fewer characteristics in the current investigation. Furthermore, it was discovered that the alpha band seems to have more characteristics than the beta and delta bands. In EEG examinations of depressed people, the most common area which has been observed to be changed is the alpha frequency spectrum.

## 5. Conclusion

An IACO-SVM algorithm is presented in depth in this study. The suggested framework is comprised of two steps: FS and categorization. In summary, research has discovered an aberrant frontal alpha asymmetry that impairs behavioral approach avoiding inclinations, which can lead to sadness. Finally, changes in anxiety symptoms may be due to greater activity in the beta frequency spectrum, but this may vary across the patients in this study. This could explain why the beta frequency spectrum has fewer features than the alpha frequency range. Taken together, the criteria used in this study are usually complementary to studies that demonstrated group-level differences between UD and BD. Anyway, the final goal of studying the differences between psychiatric conditions is to find molecular markers that seem to be unique to every condition. Univariate statistics, in particular, avoid making conclusions on an individual basis, making them less helpful for clinical usage. In this case, SVM-based techniques are critical since they allow for individual-based predictions as well as the specificity and sensitivity levels of the selected variables.

## Figures and Tables

**Algorithm 1:**
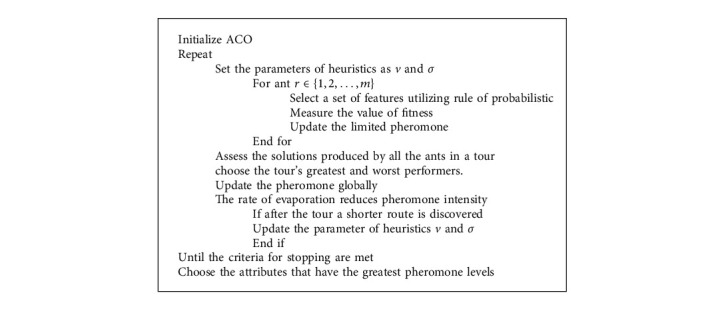
ACO algorithm.

**Table 1:** Setup of parameters for GA.

Sl.no	Parameters	Description
1	Scale	0.8
2	Crossover point	2 points
3	Rate of mutation	0.2
4	Values of fitness	Classification of accuracy
5	Patients	String (0,1)

**Table 2:** SVM classifier utilizing three distinct kernel types.

Types of kernels	Accuracy (%)
Polynomial	58.42
Linear	56.42
Radial basis function (RBF)	62.38

**Table 3:** FS algorithm performance.

FS techniques	No. of features	Sensitivity	Accuracy	Value of fitness	AUC
None	47	0.637	62.38	0.591	0.632
GA	25	0.8	75.25	0.725	0.778
PSO	26	0.783	73.27	0.706	0.740
ACO	26	0.837	78.22	0.751	0.780
IACO	23	0.855	80.18	0.774	0.794

**Table 4:** The average execution duration of the FS algorithm.

Techniques	Average execution duration
GA	21 minutes,13 sec
PSO	19 minutes,39 sec
Standard ACO	23 minutes,8 sec
IACO	17 minutes,12 sec

**Table 5:** FS algorithm performance.

FS technique	Quantity of features	Sensitivity	Fitness rate	Accuracy (%)	AUC
GA	25	0.800	0.725	75.25	0.772
PSO	26	0.783	0.704	78.22	0.738
ACO	25	0.837	0.751	78.22	0.778
IACO	21	0.855	0.794	80.18	0.792
None	49	0.637	0.591	62.37	0.632

**Table 6:** Selection of feature subset by using IACO.

Δ − frequency band	*γ* − frequency band	*α* − frequency band
*F*3-*F*4, *P*3-*T*5, *P*4-*T*6,	*F*3-*C*3, *F*3-*T*5, *P*4-*T*6,	*C*4-*P*4, *F*3-*P*3, *F*3-*T*5,
*F*3-*P*3,	*C*4-*P*4, *C*4-*T*6, *C*3-*C*4, *T*5-*T*6-*C*3-*P*3,	*C*3-*P*3, *F*4-*C*4-*F*6-*T*6, *P*4-*T*6, *C*3-*C*4, *P*3-*P*4

## Data Availability

The data used to support the findings of this study are available from the corresponding author upon request.
